# Olfactory deficit: a potential functional marker across the Alzheimer’s disease continuum

**DOI:** 10.3389/fnins.2024.1309482

**Published:** 2024-02-16

**Authors:** Dongming Liu, Jiaming Lu, Liangpeng Wei, Mei Yao, Huiquan Yang, Pin Lv, Haoyao Wang, Yajing Zhu, Zhengyang Zhu, Xin Zhang, Jiu Chen, Qing X. Yang, Bing Zhang

**Affiliations:** ^1^Department of Radiology, Nanjing Drum Tower Hospital, Affiliated Hospital of Medical School, Nanjing University, Nanjing, China; ^2^Institute of Medical Imaging and Artificial Intelligence, Nanjing University, Nanjing, China; ^3^Medical Imaging Center, The Affiliated Drum Tower Hospital, Medical School of Nanjing University, Nanjing, China; ^4^Department of Radiology, Center for NMR Research, Penn State University College of Medicine, Hershey, PA, United States; ^5^State Key Laboratory of Pharmaceutical Biotechnology, Nanjing University, Nanjing, China; ^6^Jiangsu Key Laboratory of Molecular Medicine, Nanjing, China; ^7^Institute of Brain Science, Nanjing University, Nanjing, China; ^8^Jiangsu Provincial Medical Key Discipline (Laboratory), Nanjing, China

**Keywords:** olfaction, olfactory behavior, olfactory fMRI, Alzheimer’s disease, early assessment

## Abstract

Alzheimer’s disease (AD) is a prevalent form of dementia that affects an estimated 32 million individuals globally. Identifying early indicators is vital for screening at-risk populations and implementing timely interventions. At present, there is an urgent need for early and sensitive biomarkers to screen individuals at risk of AD. Among all sensory biomarkers, olfaction is currently one of the most promising indicators for AD. Olfactory dysfunction signifies a decline in the ability to detect, identify, or remember odors. Within the spectrum of AD, impairment in olfactory identification precedes detectable cognitive impairments, including mild cognitive impairment (MCI) and even the stage of subjective cognitive decline (SCD), by several years. Olfactory impairment is closely linked to the clinical symptoms and neuropathological biomarkers of AD, accompanied by significant structural and functional abnormalities in the brain. Olfactory behavior examination can subjectively evaluate the abilities of olfactory identification, threshold, and discrimination. Olfactory functional magnetic resonance imaging (fMRI) can provide a relatively objective assessment of olfactory capabilities, with the potential to become a promising tool for exploring the neural mechanisms of olfactory damage in AD. Here, we provide a timely review of recent literature on the characteristics, neuropathology, and examination of olfactory dysfunction in the AD continuum. We focus on the early changes in olfactory indicators detected by behavioral and fMRI assessments and discuss the potential of these techniques in MCI and preclinical AD. Despite the challenges and limitations of existing research, olfactory dysfunction has demonstrated its value in assessing neurodegenerative diseases and may serve as an early indicator of AD in the future.

## 1 Introduction

Alzheimer’s disease (AD) stands as the predominant form of dementia in the elderly, chiefly marked by memory loss, impaired language function, and logical thinking. It affects an estimated 6.7 million individuals in the United States (Rajan et al., 2021) and 32 million globally (Gustavsson et al., 2023). By 2050, the incidence of dementia is projected to double in Europe and triple worldwide (Scheltens et al., 2021), accompanied by a global socioeconomic cost estimated at $9.12 trillion (Jia et al., 2018). In addition to the classic risk gene apolipoprotein E (APOE), some genomic studies are progressively revealing core risk loci and genes associated with AD (Jansen et al., 2019; Bellenguez et al., 2022). Typical pathological features of AD include amyloid plaques, neurofibrillary tangles, and brain atrophy (Jack et al., 2019). Recent years have witnessed significant advancements in understanding the intricate pathogenesis of AD (Scheltens et al., 2021; Murdock and Tsai, 2023), diagnosis, staging (DeTure and Dickson, 2019), and the development of therapeutic drugs (Doody et al., 2014; Salloway et al., 2014). Presently, few treatments for AD have demonstrated substantive efficacy. This is due to the complex etiology, challenges in early diagnosis, and the multi-stage nature of the disease (Scheltens et al., 2021; Jucker and Walker, 2023). Consequently, an increasing number of researchers are redirecting their focus to the preclinical phase of AD for early screening of potential individuals and early intervention.

Disease progression in AD is a continuous process (Jack et al., 2018), starting with the development of significant brain pathology in asymptomatic individuals at risk. It progresses through increasing pathologic burden, eventually leading to the appearance and development of clinical symptoms. In 2018, the National Institute of Aging and the Alzheimer’s Association proposed a purely biological definition of AD based on core biomarkers. This diagnostic framework emphasizes the central role of amyloid-beta protein (Aβ) and hyperphosphorylated tau protein in the AD continuum (Jack et al., 2018). The cognitive staging of the AD continuum, encompassing both Aβ and tau abnormalities, includes three primary stages: preclinical, MCI, and dementia (Jack et al., 2018). The preclinical stage of AD characterizes a phase where AD pathology is present, yet objective cognitive performance on standardized tests remains within the normal range. This stage encompasses subtle cognitive decline alongside subjective cognitive decline (SCD) (Sperling et al., 2011; Jessen et al., 2014). SCD may serve as a symptomatic indicator of preclinical AD, reflecting cognitive decline in individuals at this early stage (Jessen et al., 2014). Given the current lack of outcome benefits from various clinical trial interventions for individuals who have progressed to the dementia phase of AD (Doody et al., 2014; Salloway et al., 2014), there is an urgent need for early screening and intervention in at-risk populations.

Beyond genetic, pathologic, cognitive, and imaging evaluators, sensory markers have received sustained attention in recent years. Of all the sensory systems in AD patients, abnormalities in the olfactory system are the most prominent (Lafaille-Magnan et al., 2017; Murphy, 2018). Olfactory disturbance is closely associated with typical manifestations of AD, including accelerated cognitive deterioration (Roberts et al., 2016; Dintica et al., 2019), neurodegeneration in the medial temporal lobe such as reduced volumes of the entorhinal cortex and hippocampus (Dintica et al., 2019), as well as dysfunction in episodic memory (Papadatos and Phillips, 2023). Advances in olfactory examination and analysis techniques, particularly the use of olfactory-related behavioral examination and olfactory functional magnetic resonance imaging (fMRI), have demonstrated their potential in the detection and evaluation in the early symptomatic and pre-dementia phase of AD (prodromal AD) (Zhang et al., 2018; Lu et al., 2019b; Zhang et al., 2019b). The olfactory functional deficits can be detected with behavior tests in all stages of the AD continuum (Sohrabi et al., 2009; Devanand et al., 2015; Roalf et al., 2017; Murphy, 2018; Jobin et al., 2021b; Tian et al., 2022; Papadatos and Phillips, 2023). Postmortem (Braak and Braak, 1991; Arnold et al., 2010; Bathini et al., 2019a; Son et al., 2021) and *in vivo* neuroimaging studies (Zhang et al., 2018; Han et al., 2019; Lu et al., 2019b; Zhang et al., 2019b; Jobin et al., 2021a) demonstrated that the brain structures in the central olfactory system are preferentially involved in early-stage AD pathology. Therefore, it is timely to review the currently published evidence for the use of olfactory measurements in the screening of prodromal and preclinical AD. This article briefly reviews the characteristics of olfactory dysfunction in the AD continuum, as well as the neuropathology and assessments in the current literature. We further discuss the key findings, current status of applications, and challenges of fMRI in the study of the AD continuum.

## 2 Olfactory deficits are prevalent in AD continuum

### 2.1 Olfactory dysfunction and contributing factors in AD

The olfactory system, being one of the most ancient sensory systems, is intricately connected to the limbic system, serving essential roles in our primal survival. Olfactory dysfunction is defined as a reduction in the capacity to detect, identify, or recall odors, potentially resulting from diverse factors such as aging (Doty and Kamath, 2014), neurodegenerative diseases, head trauma, or exposure to toxins (Daulatzai, 2015). Additional contributors to olfactory deficits encompass traumatic brain injury, rhinosinusitis, diabetes, viral infections, nasal polyps, exposure to environmental toxins, and obstructive sleep apnea (Takeda et al., 2014; Daulatzai, 2015). Olfactory dysfunction risk may also be heightened by specific medications and lifestyle factors, including alcohol consumption and smoking (Obernier et al., 2002; Ajmani et al., 2017). The olfactory system plays a consistent and pervasive role in the pathology of AD (Braak and Braak, 1991; Arnold et al., 2010; Bathini et al., 2019a; Yoo et al., 2020; Son et al., 2021). AD is a multifaceted disorder involving diverse systems, pathways, and molecules, encompassing clinical, physiological, tissue, genetic, and environmental components (Scheltens et al., 2021). Likewise, contributors to AD-related olfactory impairment extend across different levels, including genetics (Graves et al., 1999), proteins (Bathini et al., 2019a), synaptopathy (Stern et al., 2004; Palop et al., 2007; Wesson et al., 2011), brain tissue atrophy (Chen et al., 2022a), neural circuitry impairments (Yan et al., 2022), and abnormalities in the activation of higher olfactory-related cortical networks (Lu et al., 2019a,b). In terms of genetic factors, the APOEε4 allele has been reported to exhibit a significant correlation with impaired olfactory function and an elevated risk of AD (Graves et al., 1999; Olofsson et al., 2010). Impaired odor identification is linked to neurodegenerative biomarkers, encompassing atrophy of the entorhinal cortex and hippocampus, as well as amyloid burden (Growdon et al., 2015). In cognitively normal adults carrying the APOEε4 allele, odor identification has been reported to be impaired early (Graves et al., 1999; Olofsson et al., 2010), years before detectable cognitive decline on the standard measures of dementia (Murphy, 2018). The aforementioned factors may contribute to olfactory dysfunction through potential mechanisms such as hypometabolism, hypoperfusion, brain atrophy, and impaired synaptic transmission in olfactory-related areas (Daulatzai, 2015).

The olfactory bulb, piriform cortex, and entorhinal cortex, as crucial brain regions in the olfactory system, are affected early in the course of AD, exerting profound impacts on both olfactory and cognitive functions (Bathini et al., 2019a; Yan et al., 2022). Importantly, the transmission of olfactory information to the hippocampus occurs in a specific sequence, starting from the olfactory receptors, then to the olfactory bulb, and finally to the olfactory cortex. Olfactory dysfunction in AD has a negative impact on the entorhinal cortex and perforant pathway. Numerous pathological factors result in continuous synaptopathy, neurodegeneration, and synaptic loss. These factors increase the expression of pro-inflammatory cytokines and soluble Aβ, leading to functional deficits in the olfactory cortex (Stern et al., 2004; Palop et al., 2007; Wesson et al., 2011; Daulatzai, 2015). The disruption of the perforant pathway can lead to the differentiation of olfactory stimuli, potentially having adverse effects on the hippocampus (de Toledo-Morrell et al., 2007). The impairment of hippocampus-dependent memory and learning may further contribute to the early progression of AD (de Toledo-Morrell et al., 2007; Daulatzai, 2015). Olfactory impairment can be assessed by various methods, including psychophysical tests, electrophysiological examination, and neuroimaging techniques. Psychophysical tests, characterized by their convenient and cost-effective nature, have been widely applied and adopted. Several olfactory tests have been utilized to study individuals with olfactory dysfunction or those at risk of developing AD (Murphy, 2018; Su et al., 2021). Table 1 summarizes the structural basis and functional requirements of the subcomponents of the olfactory function.

**Table 1:** The definition and functional requirements of the olfactory subfunction.

	Definition	Functional requirements	Behavioral test
Odor identification	The ability to identify an odor	Olfactory detection ability and semantic memory	UPSIT; T&T; SSOT; NIH-TOIT; SDOIT; ETOC;
Odor threshold	The minimum perceived concentration of an odorant	Olfactory processing in the peripheral olfactory epithelium	T&T; SSOT; ETOC;
Odor discrimination	The ability to discriminate stimuli of different odorants	Central olfactory processing includes executive function and working memory	SSOT; MODT;

UPSIT, University of Pennsylvania smell identification test; T&T, T&T olfactometry; SSOT, the Sniffin’ sticks olfactory test; NIH-TOIT, NIH toolbox odor identification test; SDOIT, the San Diego Odor identification test; ETOC, European test of olfactory ability; MODT, match-to-sample odorant discrimination task.

### 2.2 Characteristics of olfactory dysfunction in the MCI population

Olfactory impairment is present and well-documented in MCI (Vyhnalek et al., 2015; Roberts et al., 2016; Roalf et al., 2017; Dong et al., 2023). Olfactory functional deficits have been observed through standardized behavioral testing, which can occur early in the disease process, even before clinically detectable changes in cognition and memory (Graves et al., 1999; Olofsson et al., 2010). The majority of the behavioral studies report significant declines in olfactory detection and identification abilities in MCI patients compared to age-matched healthy controls, regardless of the specific olfactory test used. This suggests that the deficits in olfactory detection and identification in AD and MCI patients are not proportional to changes in other sensory impairments (Vyhnalek et al., 2015; Roalf et al., 2017; Jobin et al., 2021b; Dong et al., 2023). Behavioral studies have shown that olfactory impairment is associated with an increased likelihood of MCI/ amnestic MCI (aMCI)/ non-amnestic MCI (naMCI), predicting faster cognitive decline (Dintica et al., 2019) and increased risk of AD progression (Daulatzai, 2015; Roberts et al., 2016; Knight et al., 2023). Among community-dwelling older adults, odor identification impairment predicted cognitive decline better than verbal episodic memory deficits (Devanand et al., 2015), whereas poorer olfaction predicted incident MCI and is associated with global and local Aβ deposition (Tian et al., 2022). Studies have shown that higher olfactory scores are associated with a reduced risk of transitioning from unimpaired cognition to MCI, and from MCI to dementia. A 1-unit increase in the olfactory score was associated with approximately 14 and 11% reduction in risk, respectively (Knight et al., 2023). This may be related to the earlier appearance of structural and functional abnormalities (Jobin et al., 2021a; Chen et al., 2022a) and faster Aβ and tau accumulation in olfactory-related regions (Murphy, 2018; Tian et al., 2022). Behavioral and brain volume measurements have shown a gradual change in individuals from normal cognition to MCI and to AD (Vasavada et al., 2015; Chen et al., 2022a), with significant atrophy of the olfactory bulb, primary olfactory cortex (POC), and hippocampus in MCI patients (Vasavada et al., 2015; Jobin et al., 2021a). These changes correlate with behavioral measures. Interestingly, the olfactory activation cortex declines more in the POC and hippocampus during the MCI phase, which may explain the more prominent olfactory deficits than other behavioral and brain volume changes at this stage (Vasavada et al., 2015). Olfactory identification has been reported to be markedly impaired in both aMCI and naMCI (Vyhnalek et al., 2015; Dong et al., 2023), which is a stronger marker for distinguishing patients with MCI from those without, and even MCI from those with other health problems (Touliou and Maglaveras, 2021).

On the other hand, olfactory dysfunction may exacerbate the cognitive impairment in individuals with MCI (Makizako et al., 2014; Daulatzai, 2015; Suzuki et al., 2021), and olfactory deficits have been reported to be associated with cognitive decline in aMCI but not in naMCI (Vyhnalek et al., 2015). A meta-analysis of studies involving MCI populations demonstrated that odor identification tests yielded larger effects than odor detection threshold or memory tests. Additionally, there may be a correlation between olfactory function and gender in individuals with MCI, with male patients potentially experiencing a greater burden of olfactory deficits, while older female subjects tend to perform better on olfactory tasks (Roalf et al., 2017). Moreover, a reduction in olfactory scores among patients with MCI has been reported to be associated with the functional metabolic representation of the olfactory cortex (Forster et al., 2010). Evidence at the electrophysiological level also corroborates previous findings of olfactory deficits in MCI and AD patients (Peters et al., 2003; Walla et al., 2011). Neuroimaging abnormalities, spanning structural, functional, and microstructural levels, are intricately associated with olfactory impairment (Cross et al., 2013; Zhang et al., 2019a; Jobin et al., 2021a), and pathological and metabolic abnormalities may represent underlying common pathways for these imaging features (Forster et al., 2010; Klein et al., 2021; Wang et al., 2023). The aforementioned evidence indicates that, beyond the current biomarkers, incorporating olfactory indicators can enhance early diagnosis, differentiate early subtypes of AD, and refine the accuracy of predicting the conversion from MCI to AD.

### 2.3 Characteristics of olfactory dysfunction in the preclinical AD population

In the ATN diagnostic framework, preclinical AD refers to cognitively unimpaired individuals with abnormal Aβ and/or tau biomarkers (Jack et al., 2018). Accumulating evidence indicates that the SCD population may be in the preclinical stage of AD, with an increased risk of future cognitive decline and progression to MCI or AD dementia (Jessen et al., 2014, 2020). SCD is defined as individuals who subjectively believe that they have memory or cognitive decline compared with the previous normal state, but objective neuropsychological tests can be within the normal range (Jessen et al., 2014, 2020). In 2014, the working group of the Subjective Cognitive Decline Initiative formally introduced the term SCD and developed a conceptual framework for researching SCD in the preclinical stage of AD (Jessen et al., 2014). Biological and neuroimaging evidence also demonstrates that individuals with SCD share similar physiological changes with AD (Sun et al., 2015; Wang et al., 2020), further indicating that SCD represents a high-risk population for AD. A meta-analysis of longitudinal epidemiological studies (long-term studies over 4 years) of the SCD population found that 26.6% of individuals could develop MCI and 14.1% could develop dementia in the future (Mitchell et al., 2014). However, individuals with SCD or MCI do not universally progress to dementia; some patients may revert to normal cognition or remain stable. Therefore, the identification of AD progression-specific behavioral manifestations and biomarkers in the SCD population will have a profound impact on the early identification and intervention of AD.

Despite the limited number of studies, available evidence indicates the presence of olfactory identification impairment in individuals with SCD. Furthermore, the severity of this impairment appears to escalate as the disease advances along the AD spectrum (Wang et al., 2021; Jobin et al., 2021b; Papadatos and Phillips, 2023). In comparison to healthy controls, individuals with SCD show a decline in olfactory identification (Jobin et al., 2021b), while the olfactory threshold is reported to be negatively correlated with SCD (Kaya and Göker, 2022). Individuals with subjective memory complaints performed significantly worse than those without subjective memory complaints in terms of olfactory discrimination, recognition, and overall olfactory function. Olfactory ability may be a potentially important biomarker for identifying memory problems in community-dwelling older adults (Sohrabi et al., 2009). Furthermore, alterations in the structure and function of olfactory-related brain regions manifest early in SCD and intensify as the disease advances through the AD continuum (Chen et al., 2022a; Papadatos and Phillips, 2023). Olfactory dysfunction is correlated with entorhinal cortex atrophy and episodic memory performance in individuals with SCD (Papadatos and Phillips, 2023). Reported associations exist between APOEε4 status and odor identification deficits in older adults without cognitive impairment. Individuals carrying the APOEε4 allele exhibit significantly lower olfactory identification ability than those without the APOEε4 allele (Murphy et al., 1998; Calhoun-Haney and Murphy, 2005). Moreover, olfactory behavioral impairments may also serve as a potential indicator for neurodegeneration and tau pathology in SCD (Risacher et al., 2017). In cognitively intact participants, impairment in olfactory identification proves to be a more effective predictor of cognitive decline than deficits in verbal episodic memory (Devanand et al., 2015). Baseline olfactory identification impairment can predict cognitive impairment after 5 years (Schubert et al., 2008). Several studies also reported no significant difference in olfactory function between SCD and normal elderly controls (Papadatos and Phillips, 2023), a possible explanation is that olfactory dysfunction in SCD patients may be relatively mild, and existing olfactory tests may not be sensitive enough to accurately measure the changes in olfactory function. In addition to more refined olfactory behavioral scales, evidence from neuroimaging will facilitate a more objective and quantitative evaluation of olfactory function. This can help identify individuals with SCD who are at risk of conversion to AD at an early stage.

## 3 Olfactory neuropathology in the AD continuum

### 3.1 Accumulation of core AD biomarkers in olfactory regions

**Figure 1:**
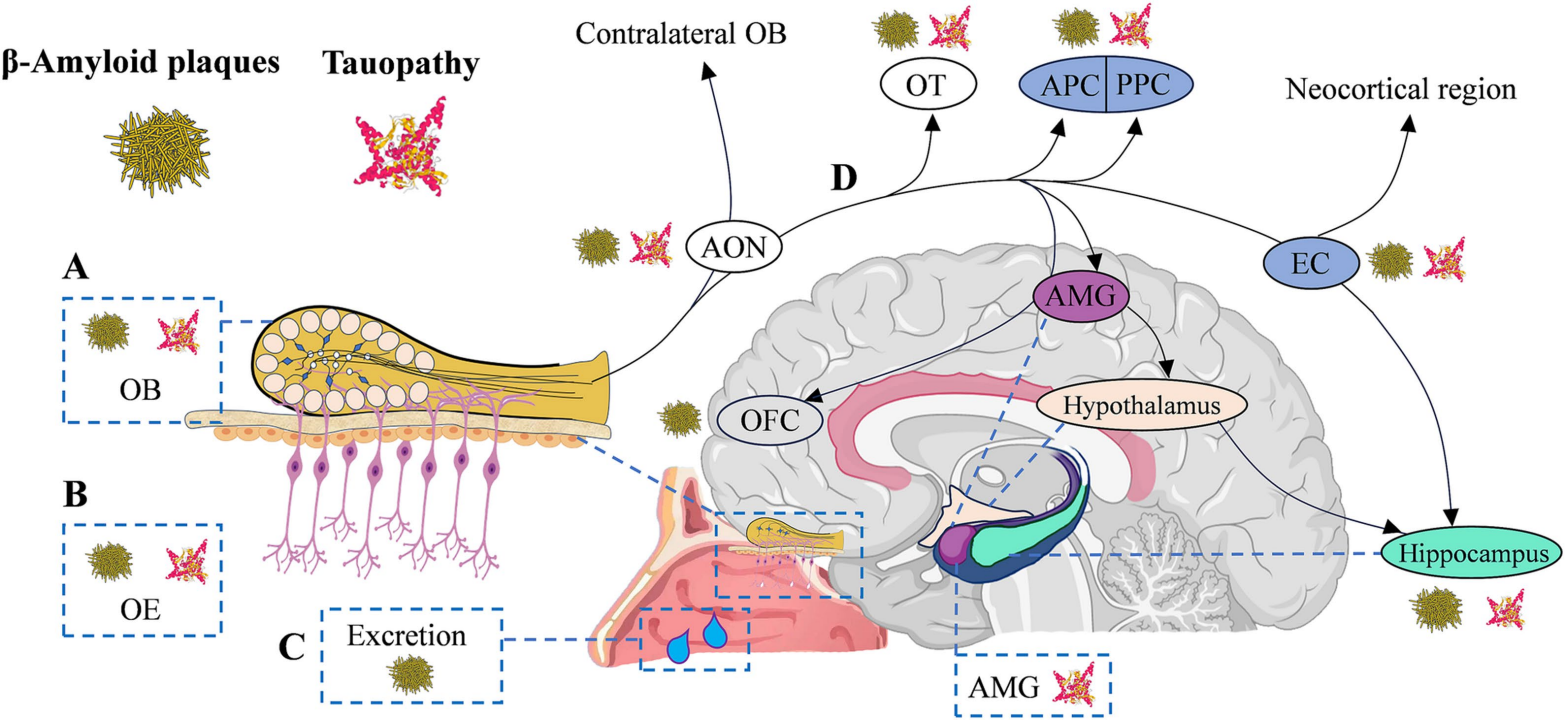
Olfactory circuitry with typical AD neuropathology and olfactory network dysfunction in AD continuum. **(A-C)** Typical AD neuropathological changes appear in the peripheral and central olfactory system of AD continuum patients, including olfactory bulb (OB, Aβ_42_ positive diffused plaques and p-tau in the glomerular region), olfactory epithelium (OE, Aβ_42_ deposition) and secretions (β-amyloid in nasal secretions), anterior olfactory nucleus (AON, β-amyloid/tauopathy), olfactory tubercle (OT, β-amyloid/tauopathy), as well as piriform cortex (PC, β-amyloid/tauopathy) and entorhinal cortex (EC, β-amyloid/tauopathy), etc. **(D)** The transmission of olfactory information to the hippocampus is sequential, proceeding from olfactory receptors to the OB, then to the AON, OT, and PC, and finally reaching the EC. EC enables olfactory input to efficiently excite hippocampal neurons through the perforated pathway. The EC is negatively impacted by olfactory dysfunction, and memory decline is upregulated by the deafferenting of the hippocampus from olfactory inputs. AD, Alzheimer’s disease; Aβ, amyloid-beta protein; AMG, amygdala; EC, entorhinal cortex; OB, olfactory bulb; OE, olfactory epithelium; OFC, orbitofrontal cortex; OT, olfactory tubercle; (A/P) PC (anterior/posterior) piriform cortex.

Olfactory neuropathology contributes to olfactory dysfunction in AD, strongly correlating with cognitive (Calhoun-Haney and Murphy, 2005) and memory dysfunction (Daulatzai, 2015; Son et al., 2021). Given that the typical pathological features of AD are amyloid plaques, neurofibrillary tangles, and brain atrophy (Jack et al., 2019), several studies have investigated the characteristics of this pathological change in the olfactory system, aiming to identify the early stages of AD through olfactory information (Talamo et al., 1991; Daulatzai, 2015; Franks et al., 2015; Son et al., 2021). As the key molecules in the pathogenesis of AD (Bloom, 2014), Aβ and/or tau protein have been detected in the form of protein pathology in patients’ olfactory system (Son et al., 2021), including olfactory epithelium (Arnold et al., 2010), olfactory bulb (Attems et al., 2014), piriform cortex (Saiz-Sanchez et al., 2015), the glomerular layer (Bathini et al., 2019b), anterior olfactory nucleus (Bathini et al., 2019b; Ubeda-Banon et al., 2020), and olfactory tubercle (Bathini et al., 2019b). Aβ in nasal secretions or nasal discharge has also been reported as a potential biomarker (Kim et al., 2019; Yoo et al., 2020) for AD and may correlate with cognitive function in patients (Yoo et al., 2020). Notably, the degree of immunoreactivity in the olfactory epithelium was reported to be correlated with averaged brain Aβ or tau pathology ratings (Arnold et al., 2010). These findings suggest that measuring these biomarkers in the peripheral olfactory system and secretions may, in part, reflect similar pathologies in the cerebral cortex in AD (Christen-Zaech et al., 2003; Arnold et al., 2010). Likewise, studies of AD mouse models have shown that β-amyloid deposition occurs earlier in the olfactory system than in any other region of the brain (Wesson et al., 2010; Son et al., 2021). There was also an association between the spatiotemporal pattern of Aβ and olfactory deficits (Wesson et al., 2010). Neuropil threads and neurofibrillary tangles of tau protein have been observed in the olfactory bulb and olfactory nerve in all cases of definite AD, as well as in many cases of probable AD, MCI, and even cognitively normal aging (Christen-Zaech et al., 2003; Attems and Jellinger, 2006). Individuals with AD spectrum diseases exhibit neurofibrillary lesions that are more severe and occur earlier in the entorhinal cortex, perirhinal, and piriform cortex (Arnold et al., 1991, 1994). These regions are involved in odor recognition and memory (Lledo et al., 2005; Walla, 2008; Glezer and Malnic, 2019), and the resulting damage ultimately may affect individuals’ olfactory behavior (Takeda et al., 2014; Murphy, 2018). Figure 1 briefly summarizes the pathological distribution of Aβ/Tau in the olfactory system and olfactory-activated network dysfunction across the AD continuum.

### 3.2 Tauopathy in the early stages of AD

From a neuropathological perspective, the olfactory system is also among the key brain regions affected earlier by core pathological markers. In the classical Braak staging, the mildest cases (Stage I-II) exhibit cortical tau pathology, primarily confined to the transentorhinal region. The *α*-projection neurons in the transentorhinal region are typically the earliest to develop neurofibrillary tangles and neuropil threads in the entire brain. Subsequently, pathology gradually accumulates in the entorhinal and other limbic systems before spreading to the neocortex (Braak and Braak, 1991). In the elderly population, primary age-related tauopathy typically concentrates in the medial temporal lobe and olfactory regions, including the olfactory bulb, transentorhinal region, and entorhinal cortex (Crary et al., 2014; Wuestefeld et al., 2023). Postmortem evidence indicates a significant correlation between early tau pathology and the thickness of the entorhinal cortex (Ravikumar et al., 2021). PET evidence suggests that the accumulation of tau protein initially occurs in the entorhinal cortex and area 35 (transentorhinal region), followed by the anterior and posterior hippocampus, area 36, and the perirhinal cortex. In individuals with intact cognitive function, an increase in tau burden is associated with localized atrophy in the entorhinal cortex, area 35, and the anterior hippocampus (Berron et al., 2021). Olfactory identification impairments correlate with the degree of tau pathology and neuroinflammation, especially in individuals exhibiting amyloid pathophysiology (Klein et al., 2021). In individuals at the early stages of AD, there is a close association among tau pathology, structural changes, and alterations in olfactory function. Therefore, abnormal tau pathology in the olfactory cortex plays a crucial role in AD-related olfactory impairment.

## 4 Olfactory dysfunction revealed by fMRI in AD continuum

### 4.1 Olfactory-related activation cortex and functional foundations

The olfactory-related activation cortex refers to the activated regions in the brain associated with the processing of olfactory information as individuals transition from smelling an odor to perceiving it. When individuals perceive odors, these regions exhibit an active state that can be visualized and studied through techniques such as fMRI. The human olfactory system has an incredible ability to distinguish more than 1 trillion olfactory stimuli (Bushdid et al., 2014). Complex olfactory information processing exists in various stages of the structural organization of the olfactory system (Walla, 2008; Gottfried, 2010; Glezer and Malnic, 2019; Zhou et al., 2019). At the first olfactory relay station, the olfactory bulb can generate a precise spatial map of olfactory information (Walla, 2008). As the largest part of the POC, the piriform cortex exhibits functional heterogeneity in the anterior–posterior axis. The posterior part of the piriform cortex encodes odor quality, while the anterior part encodes odor structure (Gottfried et al., 2006). The piriform cortex encodes odor-related information better than the OB, and the OB can encode odor recognition better than the piriform cortex, suggesting differences in the processing of olfactory information by the OB and piriform cortex (Pashkovski et al., 2020). High-throughput sequencing reveals that connectivity in the olfactory cortex is spatially organized in tripartite circuits along the anterior–posterior axis of the piriform cortex. These relatively parallel neural circuits form a division of labor, respectively processing information about perception, valence, and action in the sense of smell (Chen et al., 2022b). The amygdala responds differently to pleasant and unpleasant odors of high or low intensity, which may suggest that the amygdala encodes neither intensity nor valence *per se*, but a combination that reflects the overall emotion of the stimulus value (Winston et al., 2005). When humans are full, a majority of orbitofrontal olfactory neurons respond less to food odors, suggesting that the orbitofrontal cortex (OFC) is involved in the pleasurable or rewarding value of odors (Rolls, 2004). Likewise, as part of the secondary olfactory cortex, the hippocampus receives direct input from the EC, which is involved in odor memory and olfactory-based spatial learning (Fortin et al., 2002; Cerf-Ducastel and Murphy, 2006). During the occurrence and progression of neurodegenerative diseases, olfactory function can be affected at an early stage, garnering extensive attention in recent years for olfactory behavior and imaging examinations (Roalf et al., 2017; Murphy, 2018; Han et al., 2019; Ubeda-Banon et al., 2020; Jobin et al., 2021a).

### 4.2 Odor-evoked olfactory fMRI

The first fMRI-based olfactory imaging study commenced in 1994, where olfactory stimuli were presented, and fMRI was employed to localize the activated olfactory cortex (Koizuka et al., 1994). Over the past 30 years, as research has progressed, the application of olfactory fMRI has evolved from the analysis of normal human olfaction to the diagnosis and exploration of congenital, mental, and neurodegenerative diseases (Takeda et al., 2014; Murphy, 2018; Han et al., 2019). Traditional odor delivery methods using sniffer sticks or odor-saturated cotton are challenging to control accurately in terms of odor concentration and synchronization with image scanning. To date, specialized olfactometers have been utilized in many functional brain studies to generate olfactory stimuli. These devices typically consist of two parts: the odor transmission system and the control system. The odor transmission system consists of a positive airway pressure device, delivery tubing, and a series of odorant-containing capsules. The control system typically operates on the computer and is primarily responsible for designing and controlling the stimulus sequence. When designing an olfactory fMRI study, several technical factors, including the stimulus paradigm, odorant selection and delivery, image acquisition parameters, and data analysis methods, need to be carefully considered. These factors can significantly impact the quality and interpretability of the results, necessitating careful control and optimization to ensure the validity and reliability of the study findings.

### 4.3 Olfactory activation map in healthy subjects

The ability to distinguish between different smells is crucial for human survival, as unpleasant smells often signal danger. Specific odor stimuli can activate distinct olfactory information processing centers in humans, leading to varied olfactory experiences and behavioral decisions (Gottfried, 2010; Mori and Sakano, 2021; Torske et al., 2022). A meta-analysis of 81 olfactory-related studies revealed that brain regions significantly associated with odor activation include the piriform cortex, amygdala, insula, and OFC (Torske et al., 2022). These regions are known to play a crucial role in processing olfactory information. Examination of pleasant odors indicated that the bilateral amygdala and piriform cortex had the greatest likelihood of activation, with a more significant cluster observed in the right hemisphere. The analysis also revealed high activation peaks in the right insula, left pallidum and putamen, bilateral OFC, and central opercular cortex. In contrast, the aversive odor analysis did not show activation in the piriform cortex, unlike the other odor categories. This suggests that different brain regions may be involved in processing aversive odors compared to other odor types. The analysis of food odors indicated activation in the piriform cortex, amygdala, insula, and OFC, among others. These findings provide insights into the specific brain regions involved in processing different odor types, highlighting the complex neural mechanisms underlying olfactory perception.

Most odorants are known to be bimodal: they stimulate the olfactory nerve not only via olfactory receptors located in the upper recesses of the nasal cavity but also the trigeminal nerve (Brand, 2006; Lombion et al., 2009). Compared to olfactory stimulation, trigeminal stimulation produces additional activation in the insular cortex, temporal, cingulate, occipital, and cerebellar regions (Yousem et al., 1997; Brand, 2006). In MRI examination of olfactory stimulation, odorants with less trigeminal stimulation should be selected to attenuate trigeminal stimulated activation. Lavender oil is a potent olfactory stimulant and is often used as an odorant in olfactory fMRI studies (Wang et al., 2010; Karunanayaka et al., 2014; Zhang et al., 2018, 2019a,b) because of its little or no tendency to stimulate the trigeminal nervous system (Allen, 1936). It is generally regarded as a pleasant and familiar odorant, and in healthy people, lower concentrations of lavender oil (0.10%) can cause potent activation of olfactory-related brain regions, including the POC, insula, OFC, and hippocampus (Wang et al., 2010). Additionally, the use of lavender oil in aromatherapy helps reduce the anxiety levels of individuals undergoing MRI scans (Wen et al., 2023), making it an effective choice of odorant in olfactory MRI examinations.

### 4.4 Brain olfactory-related network detected by fMRI

The olfactory network (ON) refers to the complex network of the co-activation mode involved in olfactory information processing, including olfactory receptors, olfactory bulb, olfactory cortex, and other related brain regions. During odor-evoked tasks, the activated brain area associated with odor detection, recognition, and information processing is defined as the ON. In the resting state, characterizing the ON requires the use of *a priori* templates or regions of interest (ROI) and hypothesis-driven research methods.

Studying the ON can enhance our understanding of the processing and perception mechanisms of olfactory information. Currently, fMRI-based ON construction and research methods used in fMRI studies (Georgiopoulos et al., 2019; Arnold et al., 2020; Carlson et al., 2020; Tu et al., 2023) mainly include functional connectivity analysis based on ROI, independent component analysis technology, and complex brain network analysis based on graph theory. Functional connectivity refers to the evaluation of the temporal correlation of neural activity among anatomically separated brain regions (either voxel-based or ROI-based) (Damoiseaux et al., 2006). The main brain regions contained in the ON identified by fMRI mainly include the anterior insula, hippocampus, piriform cortex, amygdala, OFC, precuneus, and thalamus (Seubert et al., 2013; Karunanayaka et al., 2017a; Georgiopoulos et al., 2019; Carlson et al., 2020). Arnold et al. revealed four characteristics of the ON: modular composition, small-world properties, subnetwork integration, and phylogenetic conservation (Arnold et al., 2020). The ON is composed of three subnetworks, including the sensory, limbic, and frontal subnetworks, which exhibit robust small-world properties. These subnetworks are highly integrated and linked through hub nodes in the anterior insula and amygdala.

### 4.5 Abnormal connection and activation between between ON and DMN in the AD continuum

Among the numerous brain networks, the Default Mode Network (DMN) stands out as one of the most extensively investigated in AD research. The intrinsic activity of the DMN is primarily associated with functions such as introspection, self-awareness, social cognition, and emotional processing (Raichle, 2015). In AD spectrum disorders, the weakened connectivity of the DMN is closely linked to declines in various cognitive functions. Additionally, some studies have revealed anomalies in the connectivity between the DMN and ON in individuals within the AD spectrum. Studies based on independent component analysis showed that, in the odor-visual association paradigm, both the experimental conditions of odor + vision and vision alone could induce DMN inactivation. In contrast, visual trials under the odorless paradigm did not induce consistent DMN inactivation. These results suggest that directional connectivity between the DMN and the ON differs significantly between the odor + vision and vision-only trial conditions (Karunanayaka et al., 2017b). These findings support a strong interplay between the DMN and the ON and underscore the role of the DMN in task-evoked brain activity and behavioral responses during olfactory processing. AD-specific pathological damage can involve the hippocampus and the POC in ON at an early stage and is closely related to the olfactory and memory deficits of patients. Lu et al. once reported significant differences in functional connectivity between the ON and the right hippocampus (DMN) at rest across different stages of MCI (early MCI > late MCI) (Lu et al., 2019a). These findings suggest that ON and DMN are functionally connected via the hippocampus and functional connectivity between ON and hippocampus is a sensitive marker of AD progression and may precede loss of gray matter volume (Lu et al., 2019a,b). Notably, due to its straightforward scanning paradigm, resting-state fMRI has been extensively used in cognitive neuroscience research. However, it should be noted that at the resting-state level, functional connectivity or brain activations related to olfaction are based on prior templates, blind source separation, as well as hypothesis-driven statistical tests, lacking direct signal measurements related to olfactory activation or odor perception processes. Compared with resting-state fMRI alone, olfactory task-based fMRI may further complement and effectively validate conclusions drawn from resting-state studies.

### 4.6 Functional deficits of olfactory cortex revealed by fMRI in AD

Previous task and task-free MRI studies have revealed olfactory-related brain changes in AD patients at structural, functional, and graph-based levels (Thomann et al., 2009; Wang et al., 2010; Feng et al., 2021; Steffener et al., 2021; Zhu et al., 2023). Among the brain regions involved in the processing of olfactory information, the POC (Wang et al., 2010; Vasavada et al., 2015, 2017; Steffener et al., 2021), hippocampus (Wang et al., 2010; Vasavada et al., 2015; Chen et al., 2022a), and insula (Wang et al., 2010; Zhu et al., 2023) appear to be more vulnerable in AD patients. Compared with healthy controls, AD patients mainly showed reduced activation of the above-mentioned regions, which are closely related to olfactory and cognitive performance (Wang et al., 2010; Yi et al., 2022; Zhu et al., 2023). These functional abnormalities are often accompanied by changes in the olfactory system at the structural level (Murphy et al., 2003; Thomann et al., 2009; Wu et al., 2019; Yi et al., 2022; Papadatos and Phillips, 2023). Steffener et al. (2021) reported that odor-detection task-related signals were reduced in all regions of the POC in AD patients, with abnormal activity in the entorhinal cortex being the most evident among the groups. Similarly, significant POC and hippocampal atrophy and decreased activation volume can be found in both AD and MCI subjects, which are correlated with behavioral measures (Vasavada et al., 2015). Vasavada et al. also found that compared to the normal group, both the MCI and AD groups showed significantly reduced POC activation in both odor and odorless conditions (Vasavada et al., 2017). Zhang et al. used olfactory fMRI with different odor concentrations to evaluate the function of the POC and found that the POC activation pattern in the control group showed olfactory adaptation at higher concentrations, while AD patients not only showed increased olfactory thresholds but also lacked olfactory adaptation (Zhang et al., 2019a). In summary, fMRI can effectively identify the olfactory-related functional changes in AD patients, and the activation changes in the POC region are the most obvious and robust. These changes are closely related to olfactory and cognitive performance and are often accompanied by atrophy at the structural level.

### 4.7 Characteristics of olfactory fMRI changes in MCI and SCD population

Currently, there have been limited functional MRI studies on olfaction in the early-stage AD population, but studies are starting to reveal the brain regions that are impacted during important olfactory tasks. Compared to healthy controls, reduced POC activation under odorant and non-odor conditions has also been reported in MCI (Vasavada et al., 2017). Zhang et al. reported that most of the MCI subjects in their study showed odor adaptation disturbance in the POC region (Zhang et al., 2019a). These findings suggest that the POC activation patterns displayed by olfactory fMRI at different concentrations can be used to evaluate olfactory function, which is of great significance for the detection of MCI. Particularly, a significant deficit in olfactory identification was observed in aMCI patients compared to the control group, with the most prominent impairment being in the identification of pleasant and neutral odors. Moreover, aMCI patients rated pleasant and neutral odors much lower than the control group (Tu et al., 2023). Although the existing evidence is very limited, some studies have begun to focus on the changes in olfactory-related brain function and structure in the SCD population (Chen et al., 2022a; Papadatos and Phillips, 2023). Chen et al. discovered that SCD individuals exhibited a reduction in gray matter volume in olfactory-related regions, including the entorhinal cortex, piriform cortex, hippocampus, and amygdala. Additionally, the functional connectivity related to the hippocampus was significantly decreased. They also reported that as the disease progressed from SCD, MCI to AD, the reduction in gray matter volume in olfactory-related regions was accompanied by increasingly severe functional connectivity reduction. It is worth noting that the extent of gray matter volume reduction in the hippocampus was able to differentiate between the three groups (Chen et al., 2022a). Similar progressive changes in the ON have also been reported at the whole-brain connectivity scale, mainly manifested as a weakening of network interactions between the ON and other brain networks (Lu et al., 2019a,b). In addition, significant correlations have also been observed between alterations in olfactory-related brain structure and function and olfactory/cognitive performance in MCI and SCD populations (Vasavada et al., 2017; Wu et al., 2019; Papadatos and Phillips, 2023; Tu et al., 2023). Olfactory behavioral and neuroimaging changes in early-stage AD population are associated with APOEε4 alleles (Graves et al., 1999; Olofsson et al., 2010; Haase et al., 2013) and early degenerative neuropathological burden (Risacher et al., 2017; Murphy, 2018) in olfactory-related regions, but the specific mechanisms are not yet clear. Further research is needed in the future to reveal the underlying mechanisms behind the relationship between olfactory impairment and the development of AD.

### 4.8 Olfactory activation deficits and cognitive abnormality in AD continuum

In the existing evidence, abnormal activation of olfactory areas specifically related to the AD continuum is mainly concentrated in the POC, hippocampus, and insula, and these functional indicators are closely related to altered cognitive function (Murphy, 2018; Lu et al., 2019a,b; Zhang et al., 2019a; Chen et al., 2022a). Activation indicators mainly include the activation mode and number of activated voxels in POC (Wang et al., 2010; Vasavada et al., 2015, 2017; Zhang et al., 2019a), the low-frequency fluctuation, regional homogeneity, and complex network indicators, as well as functional connectivity between specific brain regions (Lu et al., 2019a; Chen et al., 2022a). Behavioral measures associated with these activation abnormalities include Mini-Mental State Examination, Dementia Rating Scale 2, and memory, language, and executive measures (Wang et al., 2010; Lu et al., 2019a,b). Significant atrophy of the POC and hippocampus was found in both AD and MCI subjects and correlated with behavioral measures. While behavioral and volumetric indicators showed a gradual decline from normal cognition to MCI to AD, the volume decline in olfactory activation in the POC and hippocampus was greater in the MCI group compared with the AD group (Vasavada et al., 2015). Similarly, Wang et al. reported that odor-induced signal intensity and activation volume within the POC increased significantly with increasing odorant concentration in the AD group, but not in the control group (Wang et al., 2010). These findings suggest that the odor activation signal in the POC is sensitive to AD-related olfactory and cognitive decline and is a promising predictor for the disease progression of the AD continuum.

### 4.9 Advantages, current status, and future directions of olfactory fMRI in AD research

Traditional olfactory psychophysical tests rely on subjective feedback from participants regarding odorants. In contrast, olfactory fMRI, with its odor-related brain activation indicators, offers a higher level of objectivity. Additionally, olfactory fMRI has several advantages, including the synchronous collection of image-odor stimulus signals, high data acquisition resolution, flexibility in designing olfactory paradigms based on tasks, and subsequent co-analysis with functional-structural data as well as coordinated analysis with Aβ and Tau imaging. These advantages assist researchers in focusing more precisely on the changes in olfactory-specific brain regions in the spectrum of AD. Traditional neuropathological and MRI studies support current fMRI research comprehensively. Specifically, in the early stages of AD, olfactory-related brain regions, including the POC and hippocampus, are impacted by tau pathology (Son et al., 2021) and structural atrophy (Vasavada et al., 2015; Jobin et al., 2021a). Functionally, there is a significant attenuation in odor-related activation indicators in these regions (Wang et al., 2010; Vasavada et al., 2015; Chen et al., 2022a). However, the causal relationship between neuropathology, structural atrophy, and functional activation remains to be elucidated. Leveraging fMRI tools to further clarify the mechanisms and targets of early olfactory impairment could contribute to more targeted early interventions and regulations in the future, such as through techniques like transcranial magnetic stimulation. A detailed analysis of olfactory deficits in AD holds significant implications for understanding the early onset and progression of the disease. Olfaction, as a promising early assessment indicator, offers a non-invasive means to track disease development. Furthermore, it aids in distinguishing olfactory patterns across neurodegenerative diseases, contributes to comprehending the interplay between cognitive reserve and olfactory dysfunction, and elucidates the social and psychological impacts of AD. Significant olfactory deficits have also been reported in many other diseases comorbid with cognitive deficits, such as diabetes (Zhang et al., 2018, 2019b), Parkinson’s disease (Takeda et al., 2014; Georgiopoulos et al., 2019), and infectious diseases (Llana et al., 2023). Interestingly, in patients with Parkinson’s disease, olfactory impairment also predicts an increased risk of dementia (Takeda et al., 2014), suggesting potential shared pathogenic mechanisms, such as damage to the cholinergic neural pathway (Doty, 2017). Exploring the mechanisms of olfactory impairment and identifying associated brain targets contributes to the investigation of potential intervention strategies, such as olfactory training and neural modulation.

Currently, olfactory fMRI also faces several challenges in AD research. Firstly, the complexity and diversity of the olfactory system make fMRI data intricate and require deeper understanding. Meanwhile, the potential comorbidities related to olfactory impairment in the elderly population underscore the importance of rigorous screening for participant inclusion. Secondly, olfactory fMRI demands a relatively high level of subject cooperation, which may be compromised in AD patients due to cognitive and cooperative impairment. Additionally, there is a need for ongoing improvement in the standardization of data acquisition for olfactory fMRI and the consistency of olfactory paradigms. Future developments include leveraging advanced fMRI techniques such as high-resolution imaging and more sensitive signal detection to enhance the precision of olfactory system activity assessment. Simultaneously, integrating multimodal imaging data, such as electroencephalography, can provide more comprehensive information, advancing our understanding of early AD diagnosis and pathophysiology. Furthermore, developing specialized analysis methods and standardized procedures for olfactory fMRI is a crucial future direction to promote result reproducibility and cross-study comparisons. In the development of olfactory fMRI technology, the availability of equipment has consistently been a crucial aspect. Its widespread application in routine clinical monitoring faces various constraints, including the maintenance requirements of the equipment, the continuous updating of paradigms, and the potential financial burden on grassroots healthcare institutions that may find it challenging to afford the purchase and upkeep of these sophisticated devices. Simultaneously, the high cost of fMRI technology poses a potential limiting factor, hindering its extensive adoption on a large scale. Despite these challenges, the application of olfactory fMRI technology presents a unique opportunity for uncovering the underlying neural mechanisms of olfactory impairment.

## 5 Conclusion

Accumulating evidence suggests that subtle changes in olfaction may occur years before the appearance of AD classic clinical pathology, and declines in all aspects of olfactory function can herald the onset of the prodromal phase of AD. The olfactory dysfunction is strongly correlated with other markers of the AD prodrome. The olfactory identification function of the subjects has demonstrated a robust ability to distinguish between cognitively normal individuals and those at risk for AD in the populations of AD, MCI, and SCD. Preliminary evaluation of an individual’s olfactory function can be based on subjective or objective olfactory behavioral examinations, but the sensitivity and specificity of these examinations require further enhancement. Other olfactory functions, such as odor recognition memory and context odor identification memory, warrant future investigation. Furthermore, current evidence from structural and olfactory functional MRI indicates varying degrees of structural atrophy and odor activation abnormalities (primarily in the POC and hippocampus) in different stages of the AD spectrum. With advancements in the spatiotemporal resolution of functional MRI imaging, olfactory functional MRI may have the potential to elucidate further the neural mechanisms underlying olfactory impairment in AD. Nevertheless, future efforts should focus on mapping the progression of olfactory abnormalities to better assess the contribution of olfactory dysfunction to disease occurrence and progression. More efforts are needed to explain their potential associations with degenerative neuropathological changes, blood, and cerebrospinal fluid biomarkers to improve their sensitivity and specificity in screening preclinical AD.

## Author contributions

DL: Data curation, Investigation, Visualization, Writing – original draft, Writing – review & editing. JL: Data curation, Investigation, Visualization, Writing – review & editing. LW: Data curation, Visualization, Writing – review & editing. MY: Data curation, Visualization, Writing – review & editing. HY: Data curation, Visualization, Writing – review & editing. PL: Data curation, Visualization, Writing – review & editing. HW: Data curation, Visualization, Writing – review & editing. YZ: Data curation, Visualization, Writing – review & editing. ZZ: Data curation, Validation, Writing – review & editing. XZ: Data curation, Supervision, Validation, Writing – review & editing. JC: Conceptualization, Funding acquisition, Resources, Supervision, Writing – review & editing. QY: Conceptualization, Resources, Supervision, Writing – review & editing. BZ: Conceptualization, Funding acquisition, Resources, Supervision, Writing – review & editing.
